# Prognostic value of genetic aberrations and tumor immune microenvironment in primary acral melanoma

**DOI:** 10.1186/s12967-022-03856-z

**Published:** 2023-02-04

**Authors:** Rong Huang, Gaigai Shen, Yu Ren, Kelin Zheng, Jiayu Wang, Yan Shi, Jiani C. Yin, Lanqun Qin, Guiying Zhang, Mengke Zhao, Xinyu Su, Luqiao Li, Fufeng Wang, Yang Shao, Baorui Liu, Zhengyun Zou

**Affiliations:** 1 Department of the Comprehensive Cancer Center, Affiliated Drum Tower Hospital, Medical School, Nanjing University, Nanjing, China; 2 Nanjing Geneseeq Technology Inc., Nanjing, Jiangsu China; 3 Nanjing Drum Tower Hospital Clinical College of Nanjing University of Chinese Medicine, Nanjing, China; 4 Cancer Center, The Affiliated Changzhou No. 2 People’s Hospital of Nanjing Medical University, Changzhou, China; 5 Nanjing Drum Tower Hospital Clinical College of Nanjing Medical University, Nanjing, China; 6 Geneseeq Research Institute, Nanjing Geneseeq Technology Inc., Nanjing, China; 7 China & School of Public Health, Nanjing Medical University, Nanjing, China

**Keywords:** Acral melanoma, Tumor immune microenvironment, CDK4, M1 macrophages, Prognostic factors

## Abstract

**Background:**

Acral melanoma (AM) is the most common subtype in Chinese melanoma patients with a very poor prognosis. However, our understanding of the disease pathogenesis and molecular landscape is limited by the few studies that have been conducted. Here, we profiled the clinical characteristics, mutational landscapes and tumor immune microenvironment of AM patients to gain insights into disease characteristics and potential treatment strategies.

**Methods:**

A total of 90 AM patients were enrolled and their tissue samples were subjected to next-generation sequencing and multiplexed immunohistochemistry tests. Kaplan–Meier curves and log-rank tests were used to analyze the prognostic potential of various genetic aberrations and immune cell compositions in AM.

**Results:**

The median disease-free survival was 21.3 months and estimated median overall survival (OS) was 60 months. More advanced stages, older ages and thickness of greater than 4 mm were associated with worse prognosis in AM patients (HR = 2.57, 95% CI 1.25–5.29, *p* = 0.01; HR = 2.77, 95% CI 1.22–6.28, *p* = 0.02; HR = 3.43, 95% CI 1.51–7.82,* p* < 0.01, respectively), while patients who received post-surgical treatments had better survival (HR = 0.36, 95% CI 0.17–0.76,* p* = 0.01). The most frequently altered genes included *BRAF* (14.5%), *KIT* (16.9%), *NRAS* (12%), *NF1* (10.8%), *APC* (7.2%), and *ARID2* (6%). Copy number variations (CNV) were commonly found in *CCND1* (19.3%), *CDK4* (19.3%), *MDM2* (14.5%) and *FGF19* (12%). *CDK4* amplifications was independently associated with shorter OS in AM patients (HR = 3.61, 95% CI 1.38–9.46,* p* = 0.01). CD8 ^+^ T cells (p < 0.001) and M1 macrophages (*p* = 0.05) were more highly enriched in the invasive margin than in the tumor center. Patients with higher levels of M1 macrophage infiltration in the invasive margin derived markedly longer OS (HR = 0.43, 95% CI 0.20–0.95, *p* = 0.03). Interestingly, in *CDK4*-amplified patients, there tended to be a low level of M1 macrophage infiltration in the invasive margin (*p* = 0.06), which likely explains the poor prognosis in such patients.

**Conclusions:**

Our study provided a comprehensive portrait of the clinicopathological features, genetic aberrations and tumor microenvironment profiles in AM patients and identified candidate prognostic factors, which may facilitate development of additional therapeutic options and better inform clinical management of AM patients. Based on these prognostic factors, further studies should focus on enhancing the infiltration of M1 macrophages, especially in *CDK4*-amplified AM patients.

**Supplementary Information:**

The online version contains supplementary material available at 10.1186/s12967-022-03856-z.

## Introduction

The incidence of melanoma has been on the rise, with newly diagnosed cases increasing by 170% worldwide from 1990 to 2019 [1]. Acral melanoma (AM) is a rare and aggressive subtype that arises on the hands or feet. AM more commonly affects the Asian populations, with approximately 40% of Chinese melanoma patients having the acral subtype [2]. Complete surgical resection is considered the best option for cure in melanoma at early stages. For patients with advanced melanoma, the efficacy of surgical interventions or chemotherapy is very limited. The five-year survival rate of melanoma patients with stage I, II, III, and IV diseases were 94.1%, 44.0%, 38.4% and 4.6%, respectively [3]. Despite recent development of targeted therapy and immunotherapy, which have led to improved survival outcomes in melanoma patients [4], identification of prognostic factors might be helpful for gaining insight into the disease pathogenesis and further optimizing treatment strategies. A retrospective study of 522 Chinese melanoma patients showed that tumor stage and presence of ulceration were important prognostic factors [3]. Other clinical factors, including disease duration before diagnosis, Breslow thickness, mitotic rate, vascular invasion, regional lymph node metastasis and tumor stage, have been reported in association with AM patient survival [5]. On the other hand, limited studies have evaluated the prognostic values of molecular markers in melanoma. While some studies have indicated negative associations of prognosis with key driver alterations in *NRAS*, *BRAF*, and *KIT* in melanoma patients [2, 6–8], contradictory claim about the role of *BRAF* mutation in predicting outcomes in Korean patients with primary acral lentiginous melanoma (ALM) has also been made [9]. Even fewer studies of prognosis have been conducted in patients with AM. In such patients, the prognostic value of clinical features and genetic aberrations remains largely unknown.

In addition to clinical and genetic factors, the tumor immune microenvironment (TIME), which consists of a complex array of immune cells that carry out both anti-tumoral and immune-suppressive functions [10], has emerged as an area of intense research. The composition of immune cells in the TIME plays a critical role in tumor progression and can be exploited to forecast patient prognosis and improve treatment outcomes. It has been shown that CD8^+^ cell infiltration is associated with better survival in melanoma [11, 12]. Enrichment of CD4^+^ and CD8^+^ cells has been reported in responders to anti-PD-1 therapy in the metastatic setting [13]. Tumor-associated macrophage (TAM) is another important component of the TIME and can be mainly classified into proinflammatory M1 and anti-inflammatory M2 phenotypes. While several studies have attempted to analyze the modulating effect of TAM on melanoma development, its prognostic value has been inconclusive [14–17]. In addition, most of these studies have utilized CD68 as a marker for TAM, which is relatively nonspecific and generally used as a pan-macrophage marker. Thus, further characterization of the macrophage phenotype is also required.

Here, we performed next-generation sequencing (NGS) and multiplexed immunohistochemistry (mIHC) on baseline surgical samples from patients with primary AM to thoroughly characterize the mutational profile and immune landscape in association with patient prognosis. Through multi-dimensional analysis of clinicopathology, genetic aberrations and TIME, we investigated prognostic factors of AM, aiming to gain more insight into disease characteristics and potential treatment strategies.

## Patients and methods

### Patient population and samples collection

Ninety AM patients who admitted to Drum Tower Hospital from July 2010 and January 2021 were retrospectively included in this study. The clinicopathological information was collected, including age, sex, stage (TNM staging system, AJCC 8th Edition), Breslow thickness, local lymph node metastasis, ulceration, post-surgical treatment status and survival until the last follow-up or death. Archived formalin-fixed paraffin-embedded (FFPE) surgical-resected primary AM samples were available for all patients. And FFPE samples were confirmed by pathologists from the centralized clinical testing center before genetic testing.

### Targeted NGS and genetic analysis

Qualified samples were subjected to targeted NGS by a Clinical Laboratory Improvement Amendments-certified and College of American Pathologists-accredited clinical testing laboratory (Nanjing Geneseeq Technology Inc., Nanjing, China) using a pan-cancer gene panel (GeneseeqPrime™, Geneseeq Technology Inc.). DNA extraction, library construction, and targeted capture enrichment were carried out following standard protocols as previously described with modifications [18, 19]. In brief, FFPE samples were de-paraffinized first with xylene before genomic DNA extraction using QIAamp DNA FFPE Tissue Kit (Qiagen Cat. No. 56404) according to the manufacturer’s instructions. Genomic DNA extracted from tumor samples was qualified using Nanodrop2000 (Thermo Fisher Scientific, Waltham, MA), then quantified using the dsDNA HS assay kit on a Qubit 3.0 fluorometer (Life Technology, US) according to the manufacturer’s recommendations. Targeted NGS libraries were prepared using the KAPA Hyper Prep kit (KAPA Biosystems) with an optimized manufacturer’s protocol for different sample types. Targeted hybridization enrichment was performed as previously described [20]. According to the manufacturer's instructions, the target-enriched libraries were then sequenced on a HiSeq4000 NGS platform (Illumina).

Sequencing data was first demultiplexed and subjected to FASTQ file quality control using Trimmomatic [21]. Only data without extra nucleotide bases and passed quality control (QC above 15) were subjected to the following analyses. Raw reads were mapped to the reference Human Genome (hg19) using Burrows-Wheeler Aligner (BWA-mem, v0.7.12; https://github.com/lh3/bwa/tree/master/bwakit) [22]. Genome Analysis Toolkit (GATK 3.4.0; https://software.broadinstitute.org/gatk/) was employed to perform local realignment around the insertions/deletions (INDELs) and base quality score recalibration. Picard was used to remove PCR duplicates. VarScan2 was applied to detect single-nucleotide variations (SNVs) and INDELs. SNVs were filtered out if the mutant allele frequency (MAF) was less than 1% for tumor tissue.

### Tumor mutation burden calculation

Tumor mutation burden (TMB) was calculated as the number of non-synonymous somatic mutations, including missense, nonsense, splice-site, in-frame and frameshift mutations.

### mIHC and multispectral imaging

Immune cell subsets in the TIME were identified by mIHC and multispectral imaging. Of the cohort of ninety cases, sixty-four patients had available results of both NGS and mIHC staining. Multiplexed immunofluorescence staining was performed using PANO 7-plex IHC kit (Panovue, Beijing, China), according to the manufacturer’s instructions. T cells were identified using the CD8 marker. NK cells were identified using the CD56 marker and were divided into two categories according to the intensity of membrane staining for the CD56 protein: CD56dim (weak staining) and CD56bright (strong staining). TAMs were identified by CD68 and HLA-DR and were divided into two categories: subtype M1 (CD68+ and HLA-DR+) and subtype M2 (CD68+ and HLA-DR−). Different primary antibodies were sequentially applied, including anti-CD8 (CST70306, Cell Signaling Technology, USA), anti-CD56 (CST3576), anti-panCK (CST4545), anti-CD68 (BX50031, Biolynx, China), anti-HLA-DR (ab92511), and anti-S100 (ab52642). S100 staining was used to define the invasive margin and tumor parenchyma [12, 23]. The Mantra System (PerkinElmer, Waltham, Massachusetts, US) was used to scan the stained slides and subsequently build a single stack image. The inForm image analysis software (PerkinElmer, Waltham, Massachusetts, US) was used for the reconstruction of images of sections with autofluorescence removal, based on a spectral library for multispectral unmixing. The positive rate was defined as the ratio of the number of positive cells in total number of cells in the TC or IM, excluding necrotic cells and tissues.

### Statistical methods

Fisher’s exact tests were used to test the categorical variables between groups. Kaplan–Meier curves were used to analyze overall survival of various patient groups, and the statistical difference was analyzed using the log-rank test. A two-sided *p* value of no more than 0.05 was considered significant for all tests unless indicated otherwise (*p ≤ 0.05). All statistical analyses in this study were performed using the R Project for Statistical Computing (version 3.4.0).

## Results

### Patient overview and prognostic values of clinicopathological features

The study included 90 Chinese patients with primary AM who underwent surgery at our hospital. The clinicopathological features of the cohort were summarized in Table 1. The median age of diagnosis was 62 years (38–86 years), and 55 (61.1%) patients were females. The majority of patients presented with stage II (41/90, 45.6%) and stage III (35/90, 38.9%) disease. A high proportion of patients presented Breslow thickness > 4.0 mm (41.1%). Of the patients with known histology, ALM was the most common histological subtype, which accounted for 44.4% (40/90) of the entire cohort. Other subtypes included nodular melanoma (NM) (30, 33.3%), superficial spreading melanoma (10, 11.1%) and one case of lentigo maligna melanoma. Most lesions were located in the feet (79, 87.8%) and a few in the hands (11, 12.2%). The incidence of ulcers at the primary sites was 62.2%. None of the patients had received any anti-tumor treatment prior to surgery. Following surgical resection, sixty-six (73.3%) patients received treatments, including interferon, interferon combined with other drugs, chemotherapy, and anti-PD-1 therapy. The median follow-up time was 34 months (range: 5–114 months). The median disease-free survival (mDFS) was 21.3 months and estimated median overall survival (mOS) was 60 months. At the last follow-up, 36 patients had died.

**Table 1:** Clinical characteristics of all patients (N = 90)

Characteristics	N (%)
Age, median (range), year	62 (38–86)
Sex	
Male	35 (38.9%)
Female	55 (61.1%)
Clinical stage	
I	9 (10.0%)
II	41 (45.6%)
III	35 (38.9%)
IV	5 (5.6%)
Pathological type	
NM	30 (33.3%)
ALM	40 (44.4%)
Others^a^	11 (12.2%)
Unknown	9 (10.0%)
Breslow thickness, mm	
≤ 1.0	4 (4.4%)
1.01–2.0	16 (17.8%)
2.01–4.0	25 (27.8%)
> 4.0	37 (41.1%)
Unknown	8 (8.9%)
Primary site	
Hand	11 (12.2%)
Foot	79 (87.8%)
Ulceration status	
Yes	56 (62.2%)
No	33 (36.7%)
Unknown	1 (1.1%)
Treatment	
Yes	66 (73.3%)
No	24 (26.7%)
Mutation status^b^	
BRAFm	12 (14.5%)
NRASm	10 (12.0%)
KITm	14 (16.9%)
BRAFw&NRASw&KITw	44 (53.0%)
Survival	
Alive	54 (60.0%)
Dead	36 (40.0%)

ALM, acral lentiginous melanoma; NM, nodular melanoma; m, mutation; w, wide type

^a^Others include 10 cases of superficial spreading melanoma and 1 case of lentigo maligna melanoma

^b^Mutation status was determined according to the 83 NGS-positive patients included in the analysis

Through multivariate analysis, we found several clinical features independently associated with clinical outcome (Table 2). As expected, patients with more advanced stage diseases (stages III and IV) had worse survival compared with those with earlier stages (stages I and II)(HR = 2.57, 95% CI 1.25–5.29, *p* = 0.01). Also, most patients had derived clinical benefit from post-surgical treatments compared with those who only underwent surgery (HR = 0.36, 95% CI 0.17–0.76,* p* = 0.01). In addition, we also found that patients with Breslow thickness > 4.0 mm had worse survival than those with Breslow thickness ≤ 4.0 mm (HR = 3.43, 95% CI 1.51–7.82,* p* < 0.01). Patients with older ages also had poorer survival compared with younger patients (HR = 2.77, 95% CI 1.22–6.28, *p* = 0.02).

**Table 2:** Univariate and multivariate analyses of factors associated with overall survival

Factor	Variable	Univariate analysis			Multivariate analysis	
		HR (95% CI)		*p* value	HR (95% CI)	*p* value
Age	≥ 62 vs. < 62		3.13(1.54–6.39)	< 0.01	2.77 (1.22, 6.28)	**0.02**
Gender	Male vs. female		1.67(0.83–3.35)	0.15		
Clinical stage	III&IV vs. I&II		2.26(1.17–4.39)	0.01	2.57 (1.25, 5.29)	**0.01**
Pathological type	NM&ALM vs. other types		3.64(1.11–11.90)	0.02	3.98 (0.88, 17.97)	0.07
Breslow thickness	> 4.0 vs. ≤ 4.0		4.73(2.18–10.30)	< 0.01	3.43 (1.51, 7.82)	** < 0.01**
Primary site	Hand vs. Foot		0.18(0.02–1.31)	0.06		
Ulceration status	With vs. Without		1.58(0.76–3.28)	0.22		
Treatment	Yes vs. No		0.34(0.18–0.66)	< 0.01	0.36 (0.17, 0.76)	**0.01**
Mutation status	RAS/RAF/KITm vs. RAS/RAF/KITw		0.77(0.39–1.51)	0.44		

Bold letters represent statistical significance based on the log-rank test

ALM, acral lentiginous melanoma; NM, nodular melanoma

### Mutational landscape of AM patients

**Fig. 1:**
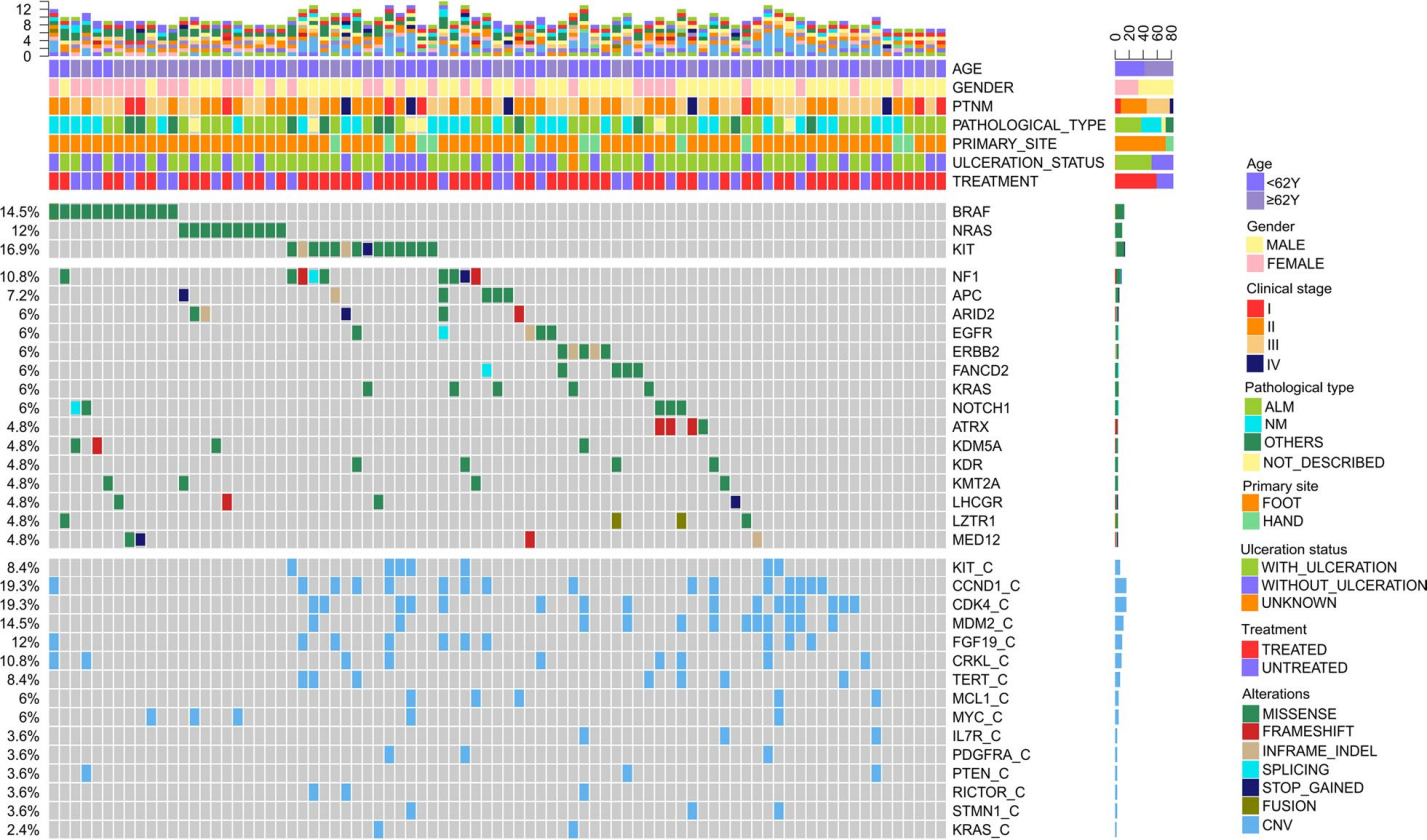
Genomic landscape of AM patients in the study (N = 83). Distributions of individual gene mutations and copy number variations in the study cohort as assessed by next-generation sequencing. Each column represents one patient. Genetic alterations were indicated in different colors according to type and clinical characteristics of each patient were shown at the top. ALM, acral lentiginous melanoma; AM, acral melanoma; NM, nodular melanoma

The top frequently altered genes in the 83 patients who had evaluable NGS results were illustrated in Fig. 1. Overall, a low TMB was observed, with a median TMB of 2.4 muts/Mb (range 0.0–15.90 muts/Mb). *BRAF*, *NRAS*, and *KIT* were most commonly altered, with respective alteration frequencies of 14.5%, 12.0%, and 16.9%. In particular, *BRAF* p.V600E and *NRAS* p.Q61K/R were the most common driver mutations, found in eight and seven patients, respectively. Missense mutations accounted for 77% of these driver mutations, and 9 (17%) copy number variations (CNV) were found in *KIT* (n = 7), *BRAF* (n = 1) and *NRAS* (n = 1). Other variant types found in these driver genes included three indel mutations (two *KIT* p.P573_D579dup, and one *BRAF* p.T599_V600delinsRE) and one fusion gene (*TRB*-*BRAF*). In addition, mutations were also commonly found in *NF1* (10.8%), *APC* (7.2%), and *ARID2* (6%). While TMB was low, the genomic landscape of AM was characterized by a high level of CNV, which was commonly found in *CCND1* (19.3%), *CDK4* (19.3%), *MDM2* (14.5%) and *FGF19* (12%). Comparing the different histological subtypes, we found that *NRAS* mutations were more commonly found in the ALM subgroup (*p* = 0.04) while *CCND1* CNVs were more enriched in the NM subgroup (*p* = 0.04) (Additional file 4: Table S1). No significant difference in TMB was observed between the ALM and NM subgroups.

### Associations of overall survival with genetic aberrations

**Fig. 2:**
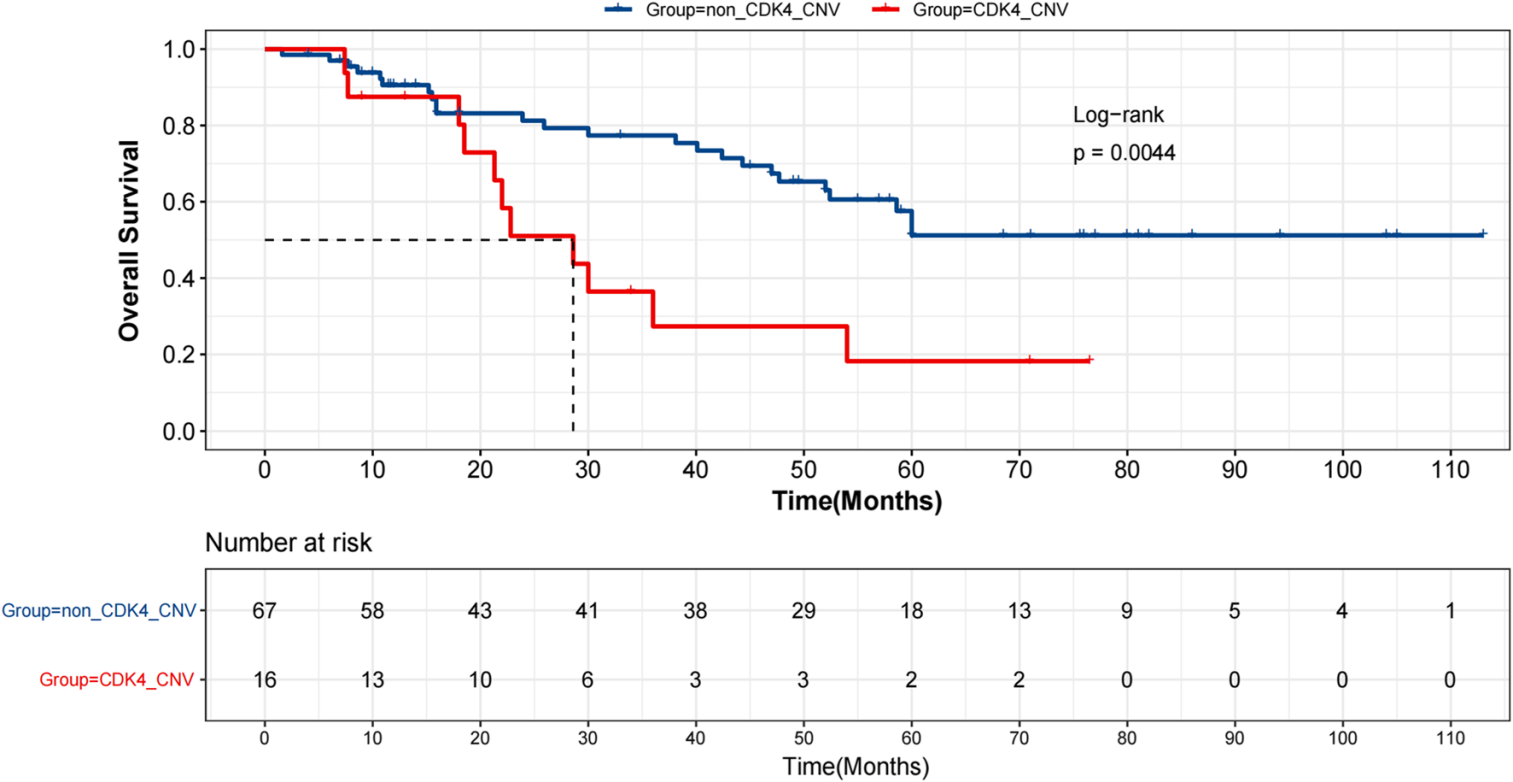
Kaplan Meier estimates of overall survival in AM patients. Survival plot comparing patients with and without *CDK4* copy number variations (*CDK4* CNV) (mOS: 28.6 m vs. not reached)

To identify putative prognostic genetic biomarkers, we performed univariate analysis of OS on genes with alteration frequencies of ≥ 3% in the study cohort. Alterations in six genes (*NSD1, KDM5A, MAP3K1, ERBB3, CDK4, TERT*) were significantly associated with OS (*p* ≤ 0.05) (Additional file 5: Table S2). Among these, *TERT* CNV was not suitable for subsequent multivariate analysis as all patients harboring *TERT* CNV were still alive at last follow-up. Multivariate analysis adjusting for clinicopathological factors on the remaining five genes revealed strong negative associations of survival with *CDK4* CNV (HR = 3.61, 95% CI 1.38–9.46, *p* = 0.01) (Additional file 6: Table S3). Patients with *CDK4* CNV exhibited markedly shorted OS compared with those without *CDK4* CNV (mOS = 28.6 m vs. not reached, *p* = 0.0044) (Fig. 2**)**. By contrast, patients carrying mutations in the common AM driver genes, including *BRAF*, *NRAS*, and *KIT*, displayed comparable survival outcomes to the wild-type patients. In addition, there was no significant survival difference comparing TMB-high and TMB-low patients (Additional file 1: Fig. S1).

### Quantitative analysis of tumor microenvironment

**Fig. 3:**
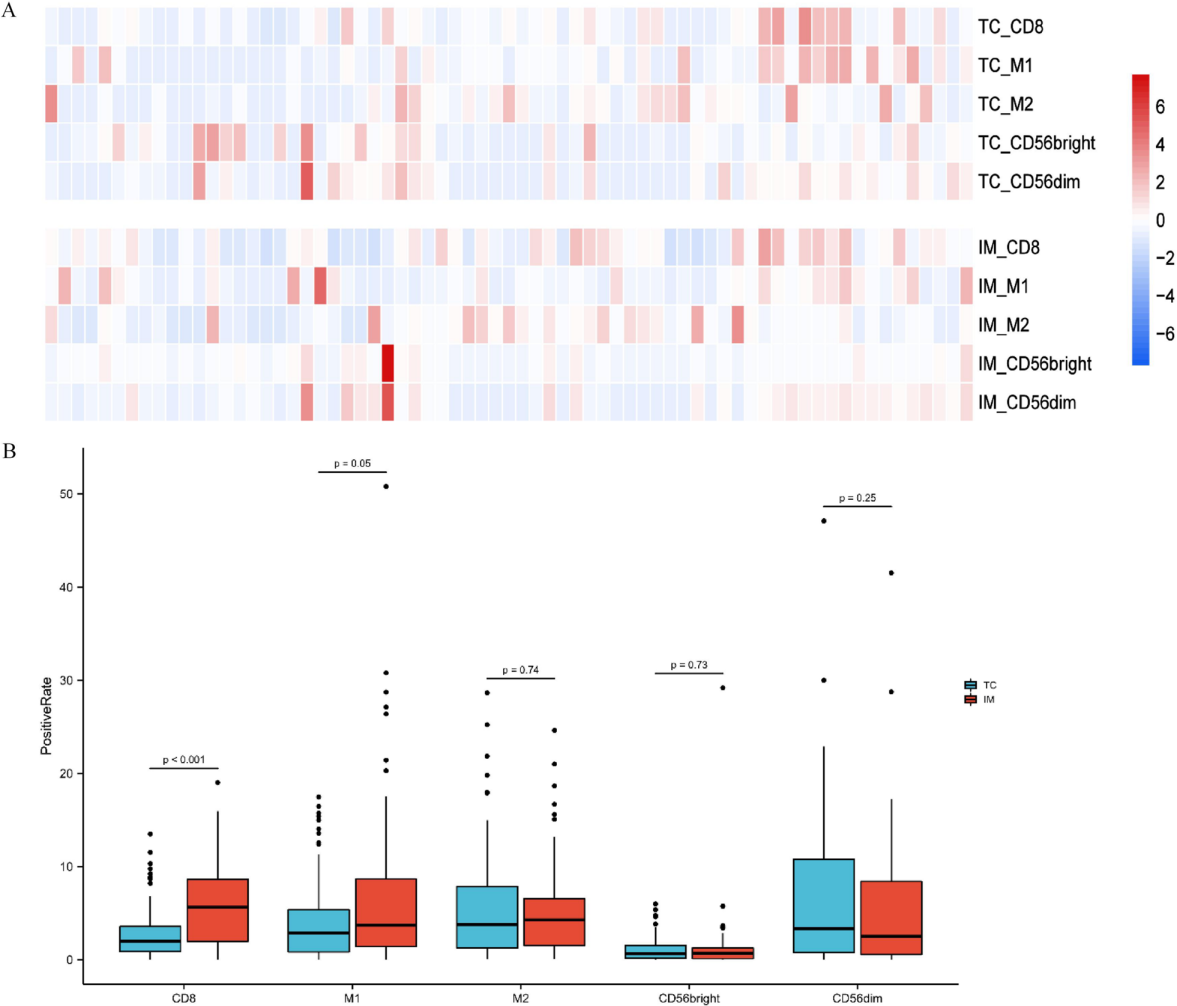
Distribution and compositions of immune cells in melanoma environment (N = 69). **A** Heatmap_Distribution of immune cell compositions in the tumor center (TC) and invasive margin (IM) in AM patients. **B** Positive rate of immune cells in TC and IM of acral melanoma patients. Wilcoxon test was used to compare the immune compositions between TC and IM

To further explore potential markers of prognosis, we analyzed 69 primary AMs for the compositions of various immune cells, including CD8^+^ T cells, M1 macrophages, M2 macrophages, CD56 bright NK cells, and CD56 dim NK cells both in the tumor center (TC) and the invasive margin (IM) by using mIHC. Overall, AM demonstrated low levels of immune infiltration (Fig. 3A). In particular, very low levels of CD56 bright cells were detected in both the TC and IM (Fig. 3B**)**. Comparing the immune compositions in the TC and IM, we found that CD8^+^ T cells (*p* < 0.001) and M1 macrophages (*p* = 0.05) were more highly enriched in the IM. No significant difference between TC and IM was found in the spatial distributions of other immune cell types, including M2 macrophages, CD56 bright NK, and CD56 dim NK cells (Fig. 3B). Significant lower levels of CD56 dim NK cells in the IM were seen in patients with primary lesions located in the hands (*p* = 0.05) compared with those with primary lesions located in the feet. Patients with driver mutations displayed higher levels of M2 macrophages both in the TC (*p* < 0.01) and IM (*p* < 0.01). Compared with other pathological types, M2 macrophages (*p* < 0.01) and CD56 bright NK cells (*p* < 0.05) were highly enriched in the patients with NM and ALM pathological type (Additional file 2: Fig. S2).

### Associations of overall survival with immune infiltration

**Fig. 4:**
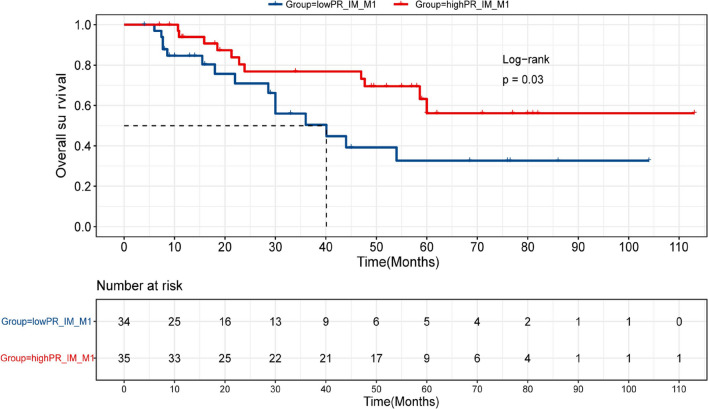
Survival analysis of M1 macrophage infiltration in the IM. Kaplan Meier estimates of overall survival in acral melanoma patients with high and low positive rate of M1 macrophages in the invasive margin (IM). mOS: not reached vs. 40.1 m

Using median positive rate of each immune cell type as the cutoff, we performed univariate analysis examining their associations with patient survival. We found that consistent with their roles on the immune system, higher levels of M2 macrophages in the TC was associated with poor prognosis (HR = 2.19, 95% CI 0.95–5.05,* p* = 0.06) (Additional file 7: Table S4), while an enrichment of M1 macrophages in the IM was correlated with favorable outcome (mOS = not reached vs. 40.1 m, HR = 0.43, 95% CI 0.20–0.95, *p* = 0.03) (Fig. 4 and Additional file 7: Table S4). Following adjusting for clinicopathological factors in multivariate analysis, high M1 macrophage infiltration in the IM remained significantly correlated with prolonged survival (HR = 0.42, 95% CI 0.81–1.01, *p* = 0.05) (Additional file 8: Table S5).

### Genetic aberrations and immune infiltration

**Fig. 5:**
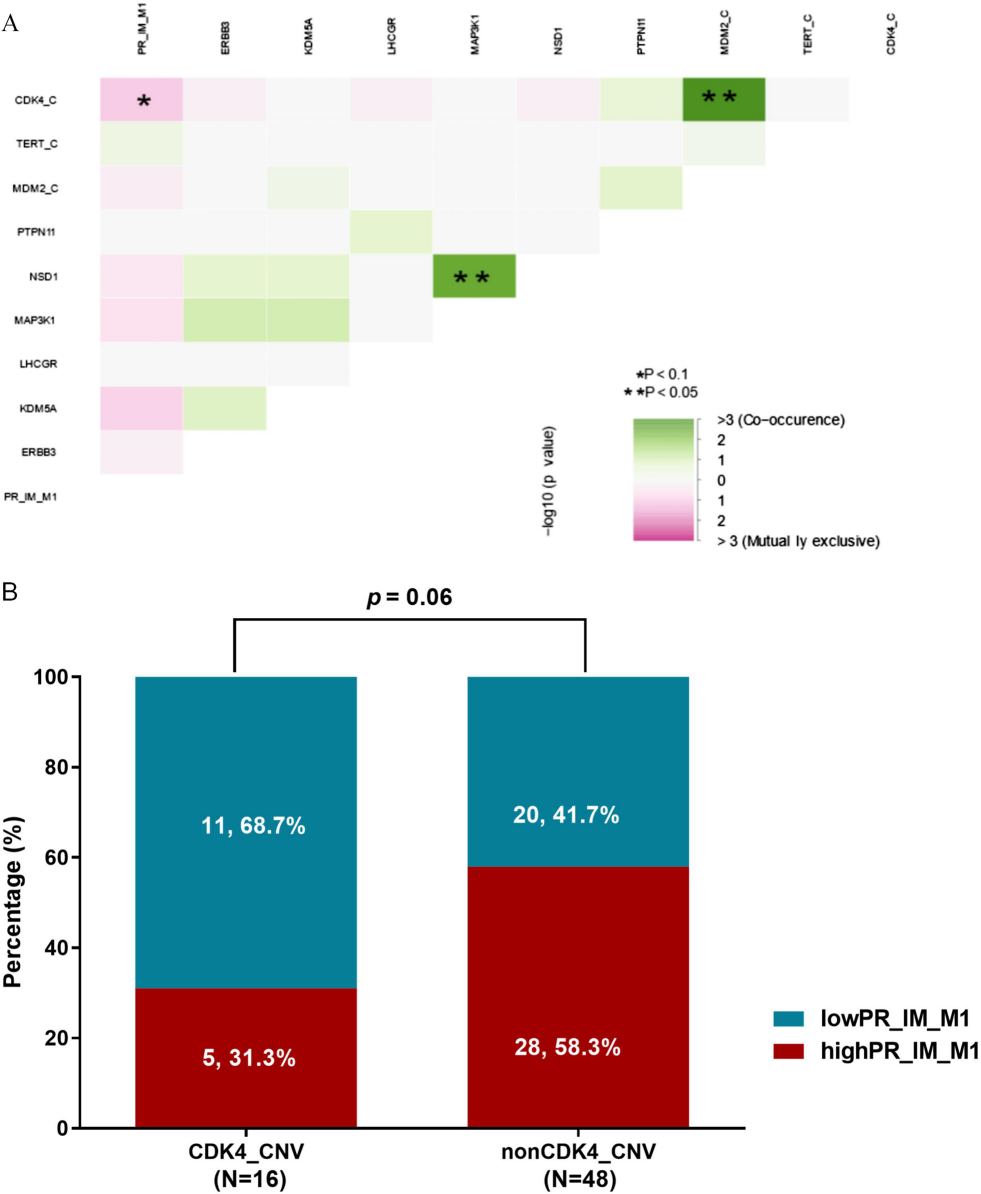
Correlation of prognosis-related genes with infiltration of M1 macrophages in the IM. **A** Influence of nine putative prognostic genetic markers on the positive rate of M1 macrophages in the invasive margin (IM). The degree of co-occurrence (green) or mutual exclusivity (pink) among factors were indicated by the color gradient. *p* value was calculated by log-rank test. **B** The correlation of *CDK4* copy number variations (*CDK4* CNV) and the positive rate (PR) of M1 macrophages in the IM. The number and proportion of patients were indicated on the top of the bar chart. *p* value was calculated by Chi-square test

Finally, we sought to determine whether there was a correlation between genetic aberrations and immune cell infiltrations in AM. Association analysis of immune cell compositions with genes (with ≥ 5% alteration frequencies), as well as TMB, revealed significant positive correlations between TMB and M1 macrophage infiltration in the TC, and CD56 dim NK cell infiltration in the IM (*p* < 0.05,* p* < 0.05) (Additional file 3: Fig. S3). We further examined the correlation of the prognosis-related genes (*p* < 0.1) with the infiltration of M1 macrophages in the IM. Interestingly, we found that AM patients with *CDK4* amplification tended to be less infiltrated with M1 macrophages in the IM (*p* = 0.06) (Fig. 5).

## Discussion

To the best of our knowledge, this is the first study to systematically explore the genetic aberrations and TIME characteristics in Chinese AM patients. Not surprisingly, patient’s age, clinical stage, Breslow thickness and postoperative treatment status had significant impact on prognosis. Interestingly, our study suggests that *CDK4* amplification may serve as a prognostic factor of AM. *CDK4* encodes a cyclin-dependent protein kinase that mediates cell cycle progression and proliferation [24]. Although previous studies have indicated a potential prognostic value of *CDK4* amplification or overexpression in several cancer types, including liposarcoma and osteosarcoma, as well as acral and mucosal melanoma [25–28], it has not been proven as an independent risk factor for melanoma prognosis [27, 28]. In our study, by adjusting for key clinicopathological features, we found that *CDK4* amplification was independently associated with unfavorable AM prognosis. Notably, no difference in survival was observed between patients with driver gene mutations (*BRAF*, *NRAS*, *KIT*) and otherwise wild-type patients. However, given the limited sample size and small number of patients with *CDK4* amplification, the prognostic role of *CDK4* in AM needs to be further validated in large prospective cohorts.

In line with previous reports, TMB was low and copy number aberrations were frequent in our AM cohort [29, 30]. Consistent with a low TMB level, which can serve a surrogate for neoantigens, we found that the AM tumors were only moderately infiltrated with immune cells. Such a unique immune profile might account for the poor response to ICIs treatment in AM patients [31]. The infiltration of CD8^+^ T cells and M1 macrophages were markedly lower in the TC than in the IM, consistent with the result reported by Gartrell et al. [11]. Their study also found a positive correlation between CD8^+^ T cells and the disease specific survival (DSS), but a negative correlation between M2 macrophages and DSS in primary melanoma. While our study demonstrated positive prognostic value of M1 macrophages in the IM in AM patients, and CD8^+^ T cell infiltration had no association with outcome, which may be due to an overall low level of CD8^+^ T cell infiltration, especially in the TC.

The impact of the genomic landscape of the tumor on the TIME has been studied in other tumor types [32, 33]. In our study, *CDK4* amplification was associated with a reduced level of M1 macrophage infiltration in the IM. Previous study has shown that selective inhibitors of *CDK4/6* induce tumor cell cycle arrest and promote anti-tumor immunity [34]. Whether *CDK4* inhibition could promote M1 macrophage infiltration in AM tumors remains unknown. In the future, more in vivo and clinical studies are needed to verify the linkage between *CDK4* and M1 macrophage as well as their roles in AM.

However, our study has several limitations. First, this is a single-center study with a relatively small sample size. Second, our study did not account for the spatio-temporal heterogeneity of immune infiltration in the tumor tissues, as composition of the TIME may undergo drastic changes during tumor evolution, which was not captured by the mIHC analysis. Finally, our study examined a small number of immune cell types, which may not reflect the complex TIME in the tumor that is generally characterized by manifold markers. Future studies are necessary to fully depict the immune cell profiles and their dynamic changes for a comprehensive understanding of the TIME.

Despite these limitations, our study provides a comprehensive analysis of AM through systematic characterization of the clinicopathological features, genetic aberrations and TIME. This is also the first study to provide clinical evidence for the positive prognostic role of M1 macrophages in AM, and to provide possible mechanistic explanations for the poor prognosis in patients with *CDK4*-amplified AM. Future studies should focus on the infiltration of M1 macrophages and explore ways to improve AM patient prognosis, likely through enhancing M1 infiltration and preventing M2 transformation in the TIME.

## Conclusions

This study systematically explores the genetic aberrations and TIME characteristics in Chinese AM patients. Our study demonstrates the negative prognostic value of *CDK4* amplification. The infiltration of M1 macrophages in the IM was also associated with poor prognostic in AM. Especially, in *CDK4*-amplified patients, there tended to be low M1 macrophage infiltration in the IM. Based on these prognostic factors, further studies should focus on enhancing M1 macrophage polarization, especially in *CDK4*-amplified AM patients.

## Supplementary Information

**Additional file 1: Figure S1.** Survival analysis of TMB. Kaplan Meier overall survival curve of acral melanoma patients with high tumor mutation burden (TMB-High) (≥ 3.5 muts/Mb) and low tumor mutation burden (TMB-Low) (< 3.5 muts/Mb). mOS: 60.0 m vs. 58.1 m.

**Additional file 2: Figure S2**. Correlation of clinicopathological features with the positive rate of immune cells. **(A)** Correlation of primary lesions with the positive rate of immune cells in the invasive margin (IM). **(B)** Correlation of driver mutations with the positive rate of immune cells in the tumor center (TC) and IM. **(C)** Correlation of pathological types with the positive rate of immune cells in the IM. *P* value was calculated by Wilcoxon test. ALM, acral lentiginous melanoma; NM, nodular melanoma.

**Additional file 3: Figure S3.** Correlation of common genetic aberrations with the positive rate of immune cells. (A, B) Correlation of genetic features with the positive rate (PR) of immune cells in the tumor center (TC) (A) and invasive margin (IM) (B). Genes with ≥ 5% alteration frequencies are included. The degree of co-occurrence (green) or mutual exclusivity (pink) are indicated by the color gradient. *: *P* < 0.05. *P* value was calculated by log-rank test.

**Additional file 4: Table S1.** Molecular differences between ALM and NM pathological subtypes.

**Additional file 5: Table S2.** Univariate analysis of genetic aberrations associated with overall survival.

**Additional file 6: Table S3.** Multivariate analysis of genetic aberrations associated with overall survival.

**Additional file 7: Table S4.** Univariate analysis of the positive rate of immune cells associated with overall survival.

**Additional file 8: Table S5.** Multivariate analysis of positive rate of immune cells associated with overall survival.

## Data Availability

The datasets generated and/or analysed during the current study are not publicly available due [REASON WHY DATA ARE NOT PUBLIC] but are available from the corresponding author on reasonable request.

## Abbreviations

ALM: Acral lentiginous melanoma
AM: Acral melanoma
CNV: Copy number variations
DFS: Disease-free survival
DSS: Disease-specific survival
FFPE: Formalin-fixed paraffin-embedded
ICIs: Immune checkpoint inhibitors
IM: Invasive margin
mIHC: Multiplexed immunohistochemistry
NGS: Next-generation sequencing
NM: Nodular melanoma
OS: Overall survival
TAM: Tumor-associated macrophage
TC: Tumor center
TIME: Tumor immune microenvironment
TMB: Tumor mutation burden
