# Supplementary figures and images for: Prognostic value of genetic aberrations and tumor immune microenvironment in primary acral melanoma
Source: J Transl Med. 2023 Feb 4;21:78. doi: 10.1186/s12967-022-03856-z (PMC9898922; doi:10.1186/s12967-022-03856-z)

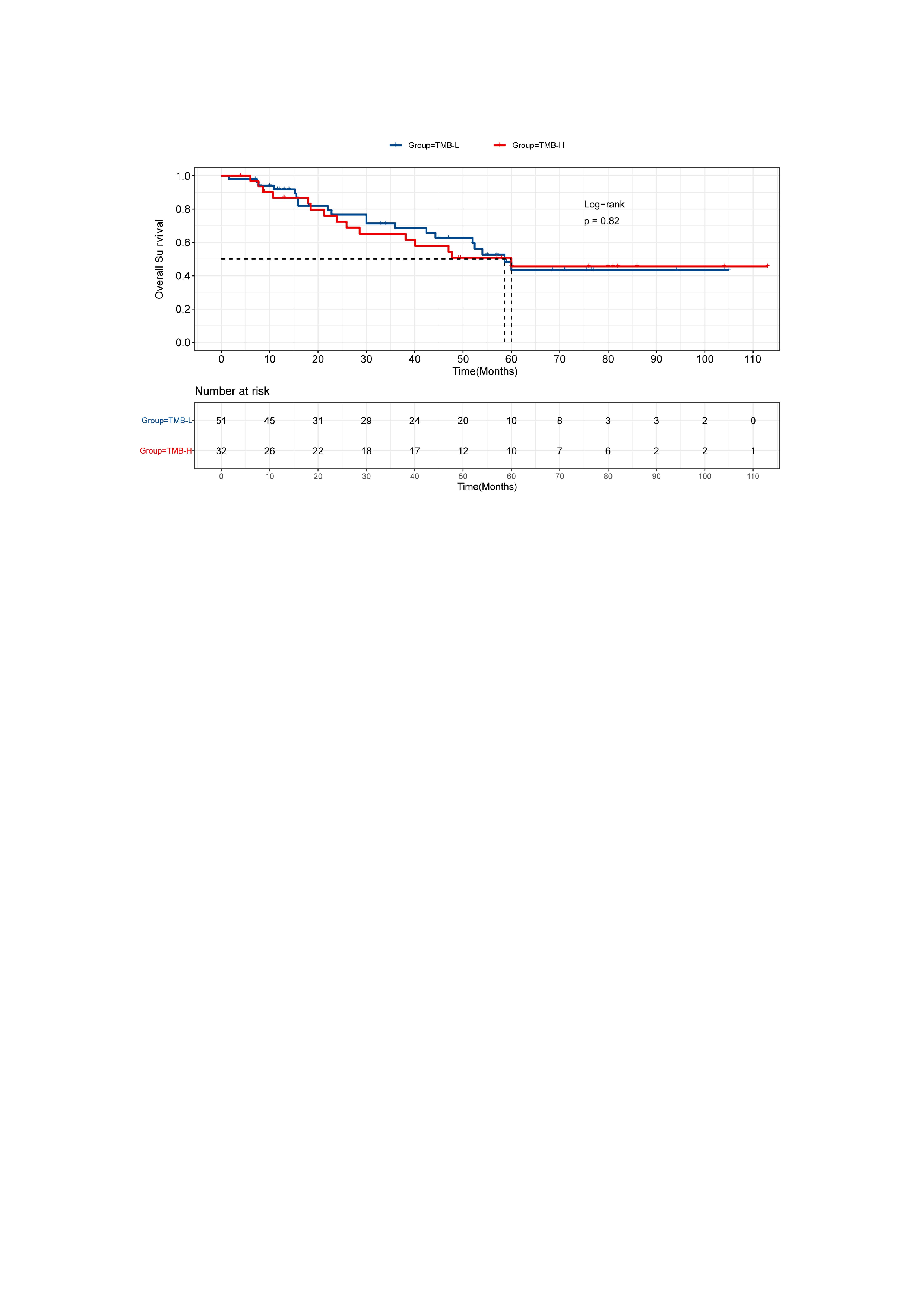

Supplement: Supplementary file 1 — Additional file 1: Figure S1. Survival analysis of TMB. Kaplan Meier overall survival curve of acral melanoma patients with high tumor mutation burden (TMB-High) (≥ 3.5 muts/Mb) and low tumor mutation burden (TMB-Low) (< 3.5 muts/Mb). mOS: 60.0 m vs. 58.1 m. [file 12967_2022_3856_MOESM1_ESM.jpg]

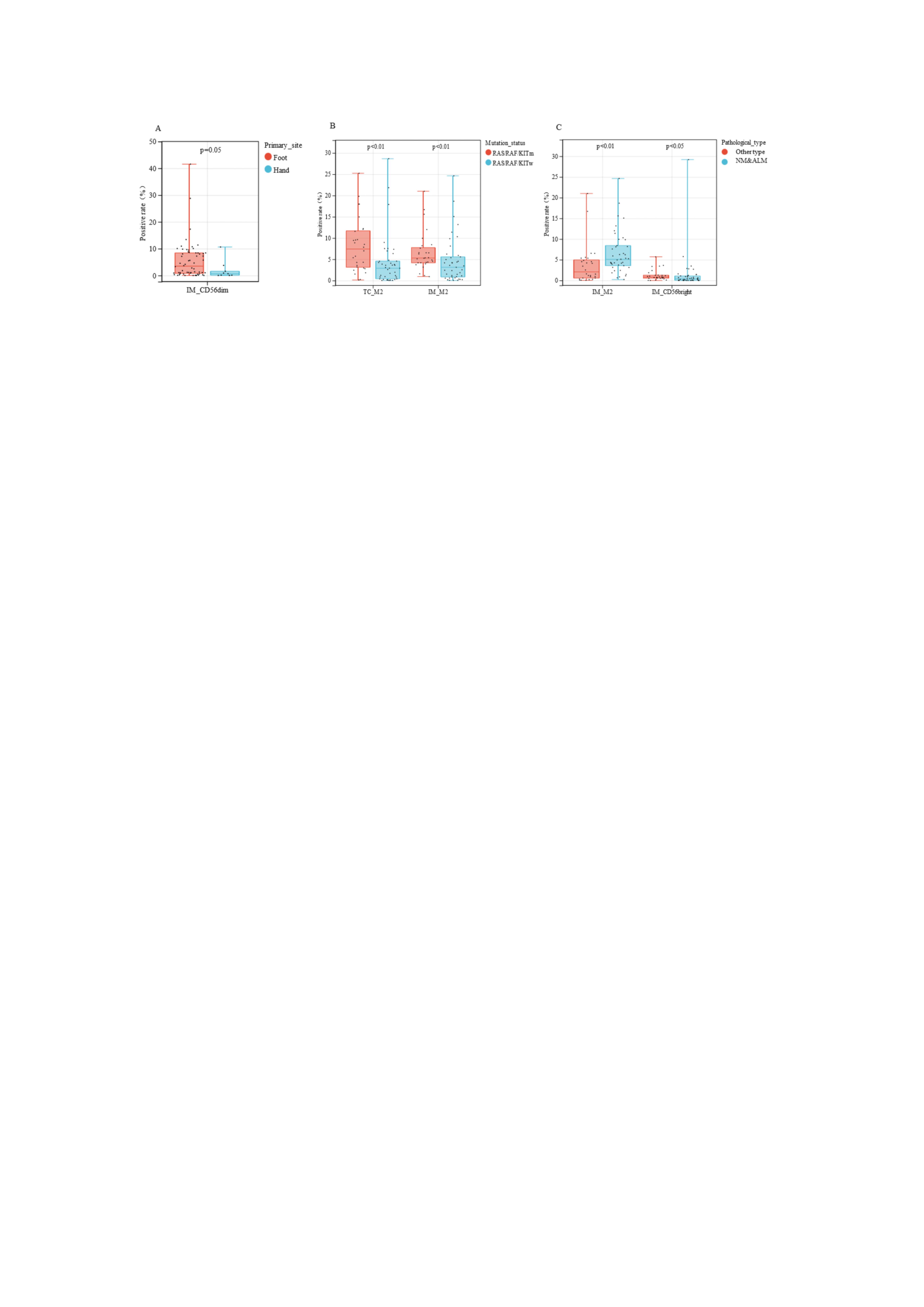

Supplement: Supplementary file 2 — Additional file 2: Figure S2. Correlation of clinicopathological features with the positive rate of immune cells. (A) Correlation of primary lesions with the positive rate of immune cells in the invasive margin (IM). (B) Correlation of driver mutations with the positive rate of immune cells in the tumor center (TC) and IM. (C) Correlation of pathological types with the positive rate of immune cells in the IM. P value was calculated by Wilcoxon test. ALM, acral lentiginous melanoma; NM, nodular melanoma. [file 12967_2022_3856_MOESM2_ESM.jpg]

**
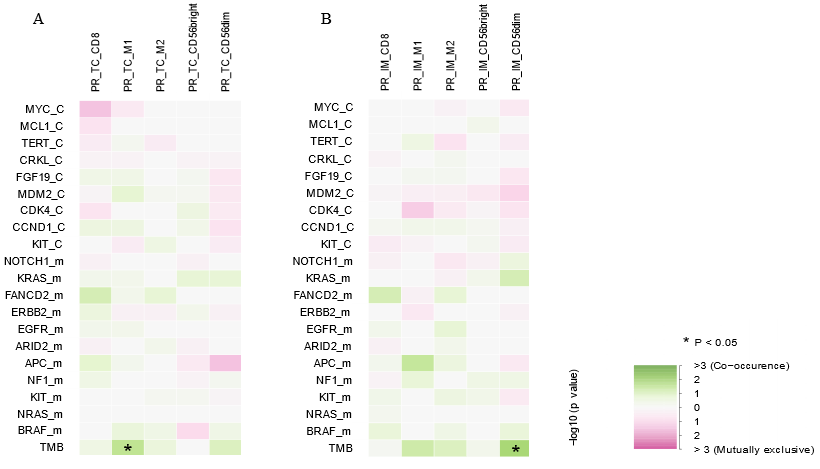
**

**Figure S3. Correlation of common genetic aberrations with the positive rate of immune cells.**

Supplement: Supplementary file 3 — Additional file 3: Figure S3. Correlation of common genetic aberrations with the positive rate of immune cells. (A, B) Correlation of genetic features with the positive rate (PR) of immune cells in the tumor center (TC) (A) and invasive margin (IM) (B). Genes with ≥ 5% alteration frequencies are included. The degree of co-occurrence (green) or mutual exclusivity (pink) are indicated by the color gradient. *: P < 0.05. P value was calculated by log-rank test. [file 12967_2022_3856_MOESM3_ESM.docx]
