# Supplementary material for: Prognostic value of genetic aberrations and tumor immune microenvironment in primary acral melanoma
Source: J Transl Med. 2023 Feb 4;21:78. doi: 10.1186/s12967-022-03856-z (PMC9898922; doi:10.1186/s12967-022-03856-z)
Supplement: Supplementary file 4 — Additional file 4: Table S1. Molecular differences between ALM and NM pathological subtypes. [file 12967_2022_3856_MOESM4_ESM.docx]

**Table S1. Molecular differences between ALM and NM pathological subtypes.**

| **Factor** | **ALM_Wild** | **ALM_Mut** | **NM_Wild** | **NM_Mut** | ***P value*** | **OR** |
| --- | --- | --- | --- | --- | --- | --- |
| NRAS | 28 | 8 | 28 | 1 | 0.04 | 0.13 |
| CCND1 CNV | 17 | 4 | 8 | 9 | 0.04 | 4.57 |

*P* value was calculated by Fisher test.

ALM, acral lentiginous melanoma; CNV, copy number variations; NM, nodular melanoma.
