# Supplementary material for: Prognostic value of genetic aberrations and tumor immune microenvironment in primary acral melanoma
Source: J Transl Med. 2023 Feb 4;21:78. doi: 10.1186/s12967-022-03856-z (PMC9898922; doi:10.1186/s12967-022-03856-z)
Supplement: Supplementary file 5 — Additional file 5: Table S2. Univariate analysis of genetic aberrations associated with overall survival. [file 12967_2022_3856_MOESM5_ESM.docx]

Table S2. Univariate analysis of genetic aberrations associated with overall survival.

| **Factor** | **Variable** | **Univariate analysis** | | **Multivariate analysis** | |
| --- | --- | --- | --- | --- | --- |
|  |  | **HR (95% CI)** | ***P* value** | **HR (95% CI)** | ***P* value** |
| Age | ≥62 vs. <62 | 3.13(1.54~6.39) | <0.01 | 2.77 (1.22, 6.28) | **0.02** |
| Gender | Male vs. Female | 1.67(0.83~3.35) | 0.15 |  |  |
| Clinical stage | III&IV vs. I&II | 2.26(1.17~4.39) | 0.01 | 2.57 (1.25, 5.29) | **0.01** |
| Pathological type | NM&ALM vs. other types | 3.64(1.11~11.90) | 0.02 | 3.98 (0.88, 17.97) | 0.07 |
| Breslow thickness | >4.0 vs. ≤4.0 | 4.73(2.18-10.30) | <0.01 | 3.43 (1.51, 7.82) | **<0.01** |
| Primary site | Hand vs. Foot | 0.18(0.02~1.31) | 0.06 |  |  |
| Ulceration status | With vs. Without | 1.58(0.76~3.28) | 0.22 |  |  |
| Treatment | Yes vs. No | 0.34(0.18~0.66) | <0.01 | 0.36 (0.17, 0.76) | **0.01** |
| Mutation status | RAS/RAF/KITm vs. RAS/RAF/KITw | 0.77(0.39~1.51) | 0.44 |  |  |

Bold letters represent statistical significance based on the log-rank test.

ALM, acral lentiginous melanoma; NM, nodular melanoma.
