# Supplementary material for: Prognostic value of genetic aberrations and tumor immune microenvironment in primary acral melanoma
Source: J Transl Med. 2023 Feb 4;21:78. doi: 10.1186/s12967-022-03856-z (PMC9898922; doi:10.1186/s12967-022-03856-z)
Supplement: Supplementary file 6 — Additional file 6: Table S3. Multivariate analysis of genetic aberrations associated with overall survival. [file 12967_2022_3856_MOESM6_ESM.docx]

**Table S3. Multivariate analysis of genetic aberrations associated with overall survival.**

| **Factor** | **HR for OS** | ***P* value** |
| --- | --- | --- |
| Age (≥62 vs. <62) | 3.68 (1.42, 9.52) | **0.01** |
| Clinical stage (III&IV vs. I&II) | 2.09 (0.91, 4.78) | 0.08 |
| Breslow thickness (>4 vs. ≤4) | 2.80 (1.16, 6.76) | **0.02** |
| Treatment (Yes vs. No) | 0.54 (0.23, 1.29) | 0.17 |
| Mutation status (Mut vs. Wild) |  |  |
| CDK4 CNV | 3.61 (1.38, 9.46) | **0.01** |
| ERBB3 | 4.36 (0.54, 35.49) | 0.17 |
| KDM5A | 1.62 (0.34, 7.76) | 0.54 |
| MAP3K1 | 5.30 (0.88, 31.89) | 0.07 |
| NSD1 | 1.81 (0.36, 9.18) | 0.47 |

Bold letters represent statistical significance based on the log-rank test.

ALM, acral lentiginous melanoma; CNV, copy number variations; NM, nodular melanoma.
