# Supplementary material for: Prognostic value of genetic aberrations and tumor immune microenvironment in primary acral melanoma
Source: J Transl Med. 2023 Feb 4;21:78. doi: 10.1186/s12967-022-03856-z (PMC9898922; doi:10.1186/s12967-022-03856-z)
Supplement: Supplementary file 7 — Additional file 7: Table S4. Univariate analysis of the positive rate of immune cells associated with overall survival. [file 12967_2022_3856_MOESM7_ESM.docx]

**Table S4. Univariate analysis of the positive rate of immune cells associated with overall survival.**

| **TIME** | **Site** | **Cut off** | **HR (95% CI)** | ***P* value** |
| --- | --- | --- | --- | --- |
| CD8^+^ T cells | tumor center | 1.98 | 1.14 (0.52~2.49) | 0.74 |
| Macrophages M1 | tumor center | 2.86 | 0.97 (0.44~2.12) | 0.94 |
| Macrophages M2 | tumor center | 3.78 | 2.19 (0.95~5.05) | **0.06** |
| CD56 bright NK cells | tumor center | 0.64 | 1.61 (0.74~3.51) | 0.23 |
| CD56 dim NK cells | tumor center | 3.33 | 1.23 (0.56~2.68) | 0.61 |
| CD8^+^ T cells | invasive margin | 5.63 | 1.17 (0.53~2.59) | 0.70 |
| Macrophages M1 | invasive margin | 3.70 | 0.43 (0.20~0.951) | **0.03** |
| Macrophages M2 | invasive margin | 4.26 | 1.11 (0.50~2.44) | 0.81 |
| CD56 bright NK cells | invasive margin | 0.67 | 0.83 (0.38~1.80) | 0.63 |
| CD56 dim NK cells | invasive margin | 2.48 | 0.97 (0.44~2.12) | 0.94 |

Bold letters represent *P* values ≤ 0.1 based on the log-rank test.
