# Supplementary material for: Prognostic value of genetic aberrations and tumor immune microenvironment in primary acral melanoma
Source: J Transl Med. 2023 Feb 4;21:78. doi: 10.1186/s12967-022-03856-z (PMC9898922; doi:10.1186/s12967-022-03856-z)
Supplement: Supplementary file 8 — Additional file 8: Table S5. Multivariate analysis of positive rate of immune cells associated with overall survival. [file 12967_2022_3856_MOESM8_ESM.docx]

**Table S5. Multivariate analysis of positive rate of immune cells associated with overall survival.**

| **Factor** | **HR for OS** | ***P* value** |
| --- | --- | --- |
| Age (≥62 vs. <62) | 4.45 (1.61, 12.31) | **<0.01** |
| Clinical stage (III&IV vs. I&II) | 2.32 (1.00, 5.41) | **0.05** |
| Breslow thickness (>4 vs. ≤4) | 0.28 (0.04, 2.12) | 0.22 |
| Treatment (Yes vs. No) | 3.64 (1.47, 9.02) | **0.01** |
| M1 macrophages in the IM (High vs. Low) | 0.42 (0.18, 1.01) | **0.05** |

Bold letters represent statistical significance based on the multivariate cox regression analysis.

ALM, acral lentiginous melanoma; IM, invasive margin; NM, nodular melanoma; TC, tumor center.
